# Vertical organic permeable dual-base transistors for logic circuits

**DOI:** 10.1038/s41467-020-18576-5

**Published:** 2020-09-18

**Authors:** Erjuan Guo, Zhongbin Wu, Ghader Darbandy, Shen Xing, Shu-Jen Wang, Alexander Tahn, Michael Göbel, Alexander Kloes, Karl Leo, Hans Kleemann

**Affiliations:** 1 Dresden Integrated Center for Applied Physics and Photonic Materials (IAPP), Technische Universität Dresden, 01062 Dresden, Germany; 2 Frontiers Science Center for Flexible Electronics, Institute of Flexible Electronics (IFE), Northwestern Polytechnical University, 710072 Xi’an, China; 3 NanoP, TH Mittelhessen, University of Applied Sciences, 35390 Giessen, Germany; 4 Dresden Center for Nanoanalysis (DCN), Center for Advancing Electronics Dresden (cfaed), Technische Universität Dresden, 01062 Dresden, Germany; 5 Leibniz-Institut für Polymerforschung Dresden e.V. (IPF), 01069 Dresden, Germany

**Keywords:** Electrical and electronic engineering, Electronic devices, Applied physics

## Abstract

The main advantage of organic transistors with dual gates/bases is that the threshold voltages can be set as a function of the applied second gate/base bias, which is crucial for the application in logic gates and integrated circuits. However, incorporating a dual gate/base structure into an ultra-short channel vertical architecture represents a substantial challenge. Here, we realize a device concept of vertical organic permeable dual-base transistors, where the dual base electrodes can be used to tune the threshold voltages and change the on-currents. The detailed operation mechanisms are investigated by calibrated TCAD simulations. Finally, power-efficient logic circuits, e.g. inverter, NAND/AND computation functions are demonstrated with one single device operating at supply voltages of <2.0 V. We believe that this work offers a compact and technologically simple hardware platform with excellent application potential for vertical-channel organic transistors in complex logic circuits.

## Abstract (web summary)

The development of vertical organic transistors with controllable threshold voltage is highly desirable for integrated circuit-based displays and sensors. Here, the authors report vertical organic permeable dual-based transistors with independently tunable on-currents and threshold voltages.

## Introduction

Organic thin-film transistors (OTFTs) can be applied for flat-panel displays^1–5^, radio frequency identification tags^6^, and sensor arrays^7,8^ owing to their excellent properties for realizing flexible, large-area electronic devices^9–11^. Organic-transistor integrated circuits for driving paper-like displays and large-area sensors have been commercially manufactured in the last decades^12,13^. Despite these advances of organic transistors, one of the major remaining issues on organic-transistor integrated circuits is the control of their threshold voltages (*V*_th_)^14,15^, i.e., the gate bias at which the transistor switches between the high current accumulation regime and the low current depletion regime. The *V*_th_ control is crucial for the design and manufacture of complicated integrated circuits. For logic gates, the *V*_th_ determines the trip point, which is the input bias at which the gate inverts the output signal^16^. For sensing applications, the *V*_th_ signifies the bias at which the largest change in current occurs, i.e., the point of the highest sensitivity^17,18^. Although there are reports in which the *V*_th_ of organic transistors is changed by the chemical channel doping of the organic semiconductors replacing the covalently bonded silane layers bearing sulfonic acid groups^19^ and by modifying the surface of the gate dielectric layers^20,21^, such approaches are still far from practical use due to the limited reliability of the manufacturing process.

To address the threshold control issue, dual-gate transistors, consisting of a single thin-film transistor with an additional second gate and second dielectric, have been developed^7,14,16,18,22^. Since the electrostatic potential and the carrier density in the whole film become a function of the second gate bias, the *V*_th_ and the off-state current can be easily tuned. Lateral-channel organic dual-gate transistors with a steeper subthreshold slope, improved carrier mobility, and an increased on/off ratio have been demonstrated^16,23^. The second gate effectively doubles the width of the channel, yielding a higher current. In addition, the opposing gate improves the gate modulation by deeper depletion of the bulk semiconductor, leading to a steeper subthreshold slope. Furthermore, the *V*_th_ shift in the transistors depends linearly on the capacitive coupling (ratio between the top and bottom gate capacitance), and the control of *V*_th_ by the second gate has been demonstrated in logic and integrated circuits^15^. Dual-gate digital integrated circuits have been used to drive actuators in a Braille sheet display and to be configured as self-contained logic gates^7^.

Vertical organic transistors have been intensively investigated in the last years^24–29^. Because of their short effective channel length, they can overcome the limits of conventional OTFTs such as a low on-current density. Organic permeable-base transistors (OPBTs)^30^, including a central electrode (base) between two outer contacts (emitter and collector) separated by organic layers, have reached a record transition frequency of 40 MHz (ref. ^31^), exhibiting great potential for switching applications. Given that the lateral-channel organic dual-gate transistors can realize high-performance logic circuits via tuning *V*_th_, thus powerful vertical stacked dual-gate/base organic transistors are highly desirable. However, to the best of our knowledge, there have been no vertical organic dual gate/base transistors reported until now. It is still challenging to precisely construct and control the dual gate/base in the vertical-channel organic transistors.

Here, we report a device concept of vertical organic permeable dual-base transistors (OPDBTs) combining two spatially separated base electrodes to simultaneously enable to change the on-currents and tune the *V*_th_. Accordingly, a high on-current density of 1.54 A cm^−2^, a large current gain of 9.2 × 10^5^, and a high transmission value of 99.998% are demonstrated in the present OPDBTs. The transmission value can be defined as differential transmission (α = d*I*_C_/d*I*_E_), the maximum differential transmission is a lower limit for the highest possible direct transmission of charge carriers through the base contact. Meanwhile, logic circuits acting as an inverter, NAND gates and AND gates operation are realized in a single-stacked OPDBT combined with a resistive load.

## Results

### Device structure and characterization

**Fig. 1:**
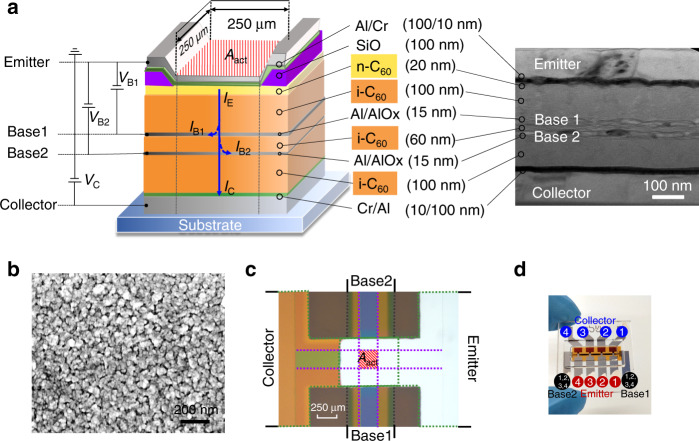
Device structure and characterization. **a** Structure schematic view of an OPDBT measured using common-emitter configuration, and cross-section TEM image of an OPDBT. **b** SEM image of a 15-nm-thick Al covered on a C_60_ layer after oxidation in ambient air. **c** Optical image of the active area in an OPDBT. The active area *A*_act_ is defined by the overlap of four electrodes: the emitter (E), base1 (B1), base2 (B2), and collector (C). An insulating layer of SiO is used to confine the active area. The current flow happens in an effective region of 250 µm × 250 µm. **d** Photograph of our OPDBTs on a glass substrate (edge length: 1 in.), including four active OPDBT pixels. With applied base1-emitter voltages (*V*_B1_) and base2-emitter voltages (*V*_B2_), electrons can pass through the base and hence reach the collector (**a**). Except for a minor leakage current into the bases *I*_B1_ and *I*_B2_ (blue dotted arrows), the emitter current *I*_E_ (solid blue arrow) can finally arrive at the collector, forming the collector current *I*_C_.

A schematic of an OPDBT is shown in Fig. 1a. The transistor consists of simple sandwich-like architecture, four parallel electrodes (gray and green) are separated by an organic semiconductor (orange). The electron conductive material C_60_ is used to realize an n-type OPDBT. All base contacts consist of a 15-nm-thick Al layer, which are covered by a thin native oxide layer formed after exposure to air. In this transistor, a 20-nm-thick layer of C_60_ doped with an efficient n-dopant W_2_(hpp)_4_ (n-C_60_, dopant concentration of 1.0 wt.%) is inserted underneath the emitter electrode in order to reduce the contact resistance and enable an Ohmic-like injection from the metal electrode^32–34^.

Cross-section transmission electron microscopy (TEM) in Fig. 1a shows the stacked layer of an OPDBT device. The top and bottom electrodes are made of Al and Cr, the C_60_ semiconductor layer with contact doping is interfacing the emitter electrode. The scanning electron microscopy (SEM) image in Fig. 1b depicts the surface morphology of the base electrode on the C_60_ film after exposure to ambient air. The base electrode is realized by 15 nm of Al evaporated at a rate of 1 Å s^−1^. A subsequent air exposure for 15 min in the dark supported the formation of a native oxide layer insulating the base. The element distribution (Al, O) in Al electrodes have been analyzed in our previous publication^35^, the AlOx thickness on the bottom and top sides of the base electrode is about 5 nm, the density of pinholes is up to 50 µm^−2^, and the diameter of pinholes is about 5 nm. The native oxide layer is sufficiently thick to act as an insulating layer, which can be seen from the following low base leakage and the high transmission values in the transfer curves. To confine the active area of the devices, two insulating layers of thermally evaporated SiO are inserted before depositing the emitter (Fig. 1a). The insulating layers have two crossed stripe-like open windows which define a quadratic active area of 250 µm in length and 250 µm in width (Fig. 1c) in OPDBTs. Figure 1d shows our OPDBT devices on a glass substrate including four transistors.

**Fig. 2:**
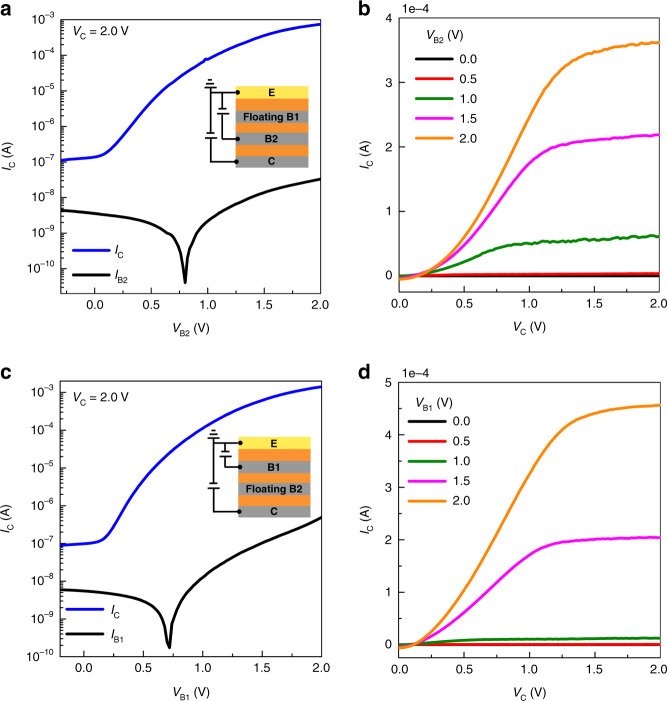
Electrical characteristics of OPDBTs measured by floating one base. **a**–**c** Transfer characteristics (**a**) and output characteristics (**b**) of OPDBTs when floating base1. **c**, **d** Transfer characteristics (**c**) and output characteristics (**d**) of OPDBTs when floating base2. The insets in **a** and **c** show the circuit connections used for the measurements. The base leakage current is negligible compared to the collector current, which underlines the high quality of our permeable-base electrodes.

Before we examine dual-base transistors, the quality of the permeable-base electrodes in an OPDBT is firstly investigated by floating one base. In the following, all electrical measurements refer to the common-emitter configuration (emitter on ground). Figure 2a, c depicts the transfer characteristics of OPDBTs measured by floating base1 and base2, respectively. Both cases show a reliable transistor behavior with on-currents of >1.19 A cm^−2^ when floating base1, and 2.25 A cm^−2^ when floating base2 at *V*_C_ = 2.0 V. Supplementary Table 1 summarizes the performance parameters of OPDBTs when floating one of the bases. Transfer curves of the OPDBTs with floating base1 reveal a transmission value of 99.996%, an on/off current ratio of 6.6 × 10^3^, corresponding to a current gain of 7.7 × 10^5^ at *V*_B1_ = *V*_C_ = 2.0 V. For floating base2, the device shows a transmission value of 99.965%, and an on/off current ratio of 1.6 × 10^4^, corresponding to a current gain of 1.4 ×  10^5^ at *V*_B2_ = *V*_C_ = 2.0 V. The current gain (*β* = *I*_C_/*I*_B_) and transconductance (*g*_m_ = d*I*_C_/d*V*_B_) curves of OPDBTs operating when floating one of the bases are shown in Supplementary Figs. 1 and 2, respectively. High current gain values indicate that the base leakage current is effectively suppressed by the native oxide layer formed on the base electrodes. Furthermore, the capacitance and phase curves versus base voltages at different supplied frequencies (1, 10, and 100 kHz) are shown in Supplementary Fig. 3. The frequency-independent depletion capacitance of the base oxide together with the phase remaining close to −90°, indicates good insulating properties of the base oxide layer^36,37^.

The output curves are measured at different base voltages of the OPDBTs for floating base1 and base2 electrodes in Fig. 2b, d, respectively. The output curves show promising behavior with a large degree of current control and saturation, even though the curves exhibit a slight nonlinear behavior at low *V*_C_ due to the contact resistance at the electrode interface^38–40^. The breakdown voltages of OPDBTs are also tested, as shown in Supplementary Fig. 4. The transistor is set into the off-state by applying one base with a voltage of −0.5 V and floating another one. By varying the collector voltage, OPDBTs can withstand a potential drop of 15.0 V. Hence, OPDBTs are sufficiently robust for logic circuit operation and all these results confirm the promising nature for our OPDBTs and thus excellent potential for applications.

**Fig. 3:**
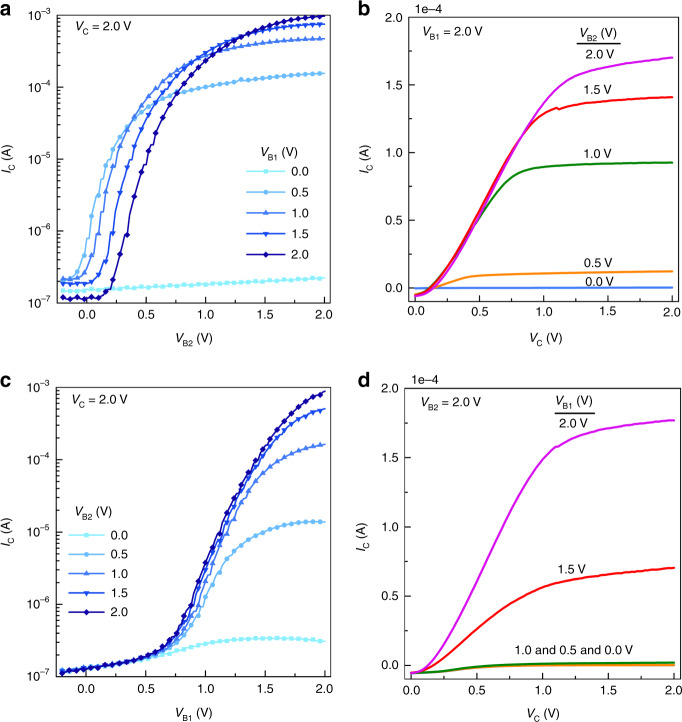
Electrical characteristics of OPDBTs. **a** Transfer curves as a function of *V*_B2_ at different *V*_B1_ of 0, 0.5, 1.0, 1.5, and 2.0 V, respectively. **b** Output curves of OPDBTs measured under constant *V*_B1_ of 2.0 V and *V*_B2_ of 0, 0.5, 1.0, 1.5, and 2.0 V, respectively. **c** Transfer curves as a function of *V*_B1_ at different *V*_B2_ of 0, 0.5, 1.0, 1.5, and 2.0 V, respectively. **d** Output curves of OPDBTs measured under constant *V*_B2_ of 2.0 V and *V*_B1_ of 0, 0.5, 1.0, 1.5, and 2.0 V, respectively.

The electrical performance of our OPDBTs for simultaneous variation of the base1 and base2 potential is shown in Fig. 3. The transfer characteristics of the OPDBTs are measured in Fig. 3a. The corresponding calculated performance parameters of the transfer curves, e.g. transmission values, on-current density, on–off ratio, the *V*_th_, transconductance (*g*_m.max._), subthreshold swing (SS) and current gain (*β*_max._), are also summarized in Supplementary Table 2. Figure 3a shows the *I*_C_-*V*_B2_ curves of the OPDBTs measured with various *V*_B1_ from 0 to 2.0 V. When *V*_B1_ = 0 V, the collector current stays at the lowest value (~10^−7^ A) in the range of the base2 sweep bias, indicating the OPDBT stays in its off-state. As V_B1_ increases from 0 to 0.5 V, the OPDBTs can be turned on. The on-current can reach ~10^−5^ A at *V*_B2_ = 2.0 V, increasing by two orders of magnitude. As the voltage of base1 further increases, the on-state current also increases sharply, the peak of on-current reaches 1.54 A cm^−2^. Also in terms of on/off-ratio, OPDBTs are not as good as single-base devices because of larger base leakage when the OPDBTs are in the off-state, leading to a lower ON/OFF ratio of 10^4^. Overall, the operation of the OPDBTs is determined not only by the base2 voltage but also by the base1 voltage.

Importantly, for the base2 sweeps, the transfer curves shift with the applied base1 bias from 0.5 to 2.0 V, so that the *V*_th_ also gradually shift from 0.04 to 0.52 V (the *V*_th_ is extracted by the point where the collector current starts to rise exponentially with the base voltage). As shown in the Supplementary Fig. 5, *V*_th_ of OPDBTs are dependent on the base1 bias during the base2 sweep. They show slight device-to-device variations measured over 61 devices. By introducing a second base layer, we can reliably control the threshold voltages of vertical organic transistors. Similarly, for base1 sweeps (Fig. 3c), transfer curves also present the on-current change and *V*_th_ shifts (from 0.68 to 0.92 V) with the applied base2 bias from 0.5 V to 2.0 V. Thus, the OPDBT allows setting the *V*_th_ of the transistor at a desired value by varying the voltages of both base electrodes. Moreover, as can be seen in Supplementary Table 2, the transmission values increase as the bias of the other control base increases from 0 to 2.0 V. Accordingly, we can modulate the charge transmission by tuning the driving voltages of base electrodes, which consequently sets the *V*_th_ of the transistors. Comparing to the single-base case when floating the additional one base, the on-current densities of 1.54 and 1.41 A cm^−2^ of OPDBTs are comparable to 1.19 and 2.25 A cm^−2^. The maximum transmission values of 99.996% and 99.965% in Fig. 2a, c are close to the maximum values of 99.998% and 99.942% in Fig. 3a, c, respectively. In addition, on/off ratios of 1.6 × 10^4^ and 6.6 × 10^3^ of OPDBTs with one floating base are also in good agreement with the values of 8.0 × 10^3^ and 7.9 × 10^3^ of OPDBTs with two bases biased. Overall, the transfer characteristics show that the extracted parameters in Fig. 3a, c match well with the ones extracted in Fig. 2a, c.

Energy diagrams relating to the four operation modes of the OPDBTs are shown in Supplementary Fig. 6. When base1 is at low potential (Supplementary Fig. 6a), the electrons can not flow to the base1 electrode, representing the off-state of the OPDBTs. The built-in potential can lead to an electric field, which would push the electrons away from the base1 region and thus prevents the current flow through the base1 electrode. When the base1 is at a high potential and the base2 at a low potential, electrons will accumulate at base1 and can not pass through base2, as shown in Supplementary Fig. 6b. Because the base electrodes are wrapped by the native oxide layer, electrons can also not flow into base1, leading to the disturbing base1 leakage^41^. Hence, the desirable result that electrons can pass through the openings in both base layers and reach the collector only happens when the base1 potential is non-zero and base2 potential larger than that of base1 (Supplementary Fig. 6c).

The base leakage currents for base1 sweeps with fixed base2 voltages from 0 to 2.0 V and for base2 sweep with fixed base1 voltages from 0 to 2.0 V are shown in Supplementary Fig. 7a, b, respectively. Compared to the base leakage in Fig. 2a, at higher base2 voltage, the base2 leakage is lower than that of the floating base1 transistor. Consequently, we obtain a large current gain of 9.6 × 10^5^ at a fixed *V*_B1_ of 2.0 V, and equal *V*_C_ = *V*_B2_ = 2.0 V in Fig. 3a, and 7.6 × 10^4^ for the base2 sweeps (Supplementary Fig. 8). However, at lower base2 voltage, the base leakage is larger than that of the floating one base transistor, which does not have an effect on the off-current level although. In Supplementary Fig. 7b, the base1 sweep shows larger base1 leakage than base2 sweep, which is attributed to the oxidation quality of base1 electrode. Furthermore, in Supplementary Fig. 9, the forward and backward sweep curves exhibit a slight hysteresis at base1 biases of 0.5, 1.0, and 1.5 V, respectively. The slight hysteresis effect which happens when V_B2_ increases from 0.5 to 1.5 V may be attributed to the trapping at the metal/semiconductor interfaces^42,43^.

Output characteristics of OPDBTs are measured and presented in Fig. 3b, d where a reasonable degree of saturation in the collector current is observed. There is a non-ideal behavior in the output curves at low *V*_C_, which is caused by the space-charge-limited current in the organic semiconductor layer^44^. A bias stress stability analysis of OPDBTs at *V*_C_ = *V*_B2_ = *V*_B1_ = 2.0 V is also performed, as shown in Supplementary Fig. 10. It can be seen that the collector, base1, and base2 currents are very stable during the stress measurement, which matches the stability of single-base OPBTs^24^. In addition, only a slight corresponding *V*_th_ shift (~0.1 V) is observed. The device-to-device reproducibility of OPDBTs is characterized in Supplementary Fig. 11 where the maximum on-current (when *V*_C_ = *V*_B1_ = *V*_B2_ = 2.0 V) distribution of 61 OPDBT devices is summarized. As indicated by the Gaussian fitting curve, OPDBTs can exhibit well reproducible and predictable device characteristics, which are critical for practical applications and determine the viability of the technology.

### TCAD simulations of OPDBTs

**Fig. 4:**
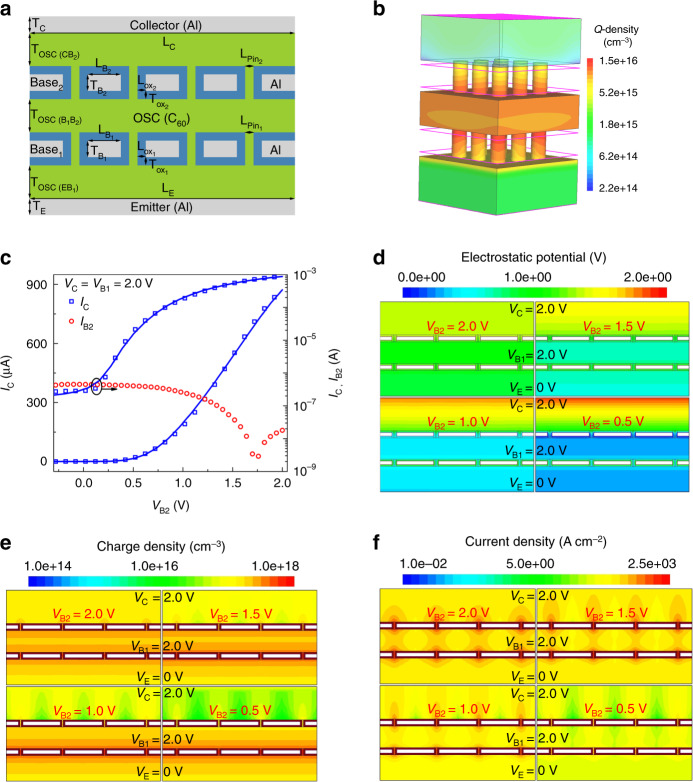
TCAD simulation of OPDBTs. **a** Schematic cross-section of the simulated OPDBT used for Sentaurus TCAD setup. **b** 3D-OPDBT structure depicting simulated charge density of the same number of pinholes in base1 and base2 *N*_Pin1_ = *N*_Pin2_ = 9, with the emitter being bottom contact and collector the top contact at *V*_B1_ = *V*_B2_ = *V*_C_ = 2.0 V. **c** Transfer characteristics: Collector current *I*_C_ from experimental data (diamonds) compared with data from TCAD simulation (solid lines), including the negligible measured base2 leakage current *I*_B2_ (circles) at *V*_B1_ = *V*_C_ = 2.0 V. **d**–**f** Electrostatic potential (**d**), charge carrier density (**e**), and current density (**f**) profiles of OPDBTs at *V*_B1_ = *V*_C_ = 2.0 V and *V*_B2_ = 0.5, 1.0, 1.5, and 2.0 V, respectively.

A schematic cross-section of the simulated structure is shown in Fig. 4a along with the device dimensions listed in Supplementary Table 3, based on fabricated OPDBT. The simulated 3D-OPDBT depicts the charge density profile at *V*_B1_ = *V*_B2_ = *V*_C_ = 2.0 V (Fig. 4b), where the carrier density through the pinholes can reach the maximum. The device current *I*_C_ flowing from emitter to collector has to pass the two base layers through the pinholes. To further elucidate the charge transport and device behavior, the measured electrical response has been compared with theoretical models. A Poole-Frenkel mobility model with a square root dependence on the electric field has been used together with the Gaussian density of states (DOS) and quantum tunneling current model. The associated device dimensions and the material parameters employed in the simulations are listed in Supplementary Table 3. The models have been used with their default parameter values except for the calibrated parameters as shown in Supplementary Table 3 (refs. ^45–47^). The simulated DC characteristics (*W* = 250 μm and *L* = 250 μm) show a very good agreement between simulations (solid lines) and the experimental measurements (diamonds) (Fig. 4c). Therefore, the calculated device characteristics mimic the measured behavior with high accuracy. Note that the base leakage current is quite small (Fig. 2) and has a negligible impact on the collector current and has been neglected in the simulation. The calibrated simulator has been taken as a reference to further investigate the performance analysis of OPDBTs.

Figure 4d–f displays the electrostatic potential, charge, and current density profiles of the simulated OPDBT in the different operation regions at *V*_B1_ = *V*_C_ = 2.0 V and different base2 voltages *V*_B2_ = 0.5, 1.0, 1.5, and 2.0 V. The working principles and the theoretical behavior of the fabricated OPDBTs have been investigated and explored based on the numerical simulation results. In Fig. 4e, when *V*_B1_ = *V*_C_ = 2.0 V, and *V*_B2_ = 0.5 V, the semiconductor exhibits a higher density of accumulated charge carriers around base1 injected from the emitter. The charge density is very low around the base2 due to lower applied potential (*V*_B2_ = 0.5 V). The carriers accumulated at base1 start to move towards base2 when *V*_B2_ increases from 1.0 to 1.5 and to 2.0 V. Therefore, the charge density is increasing around base2, resulting in a control of the collector current *I*_C_ by modulating the voltage of base2.

It is worth noting that the applied base1 voltage of 2.0 V causes carrier injection from the emitter and leads to the accumulation of charges around the base1. Thus increasing the base2 voltages *V*_B2_ = 1.0, 1.5, and 2.0 V, causes carrier migration from base1 through the pinholes toward base2 and the pinholes, allowing more carriers to pass towards the collector. Thus the device total current *I*_C_ increases (see Fig. 4f). The highest density of charge carriers in the on-state is observed in the openings (pinholes) which have a diameter of a few nanometers.

### Logic circuits realized by OPDBTs

**Fig. 5:**
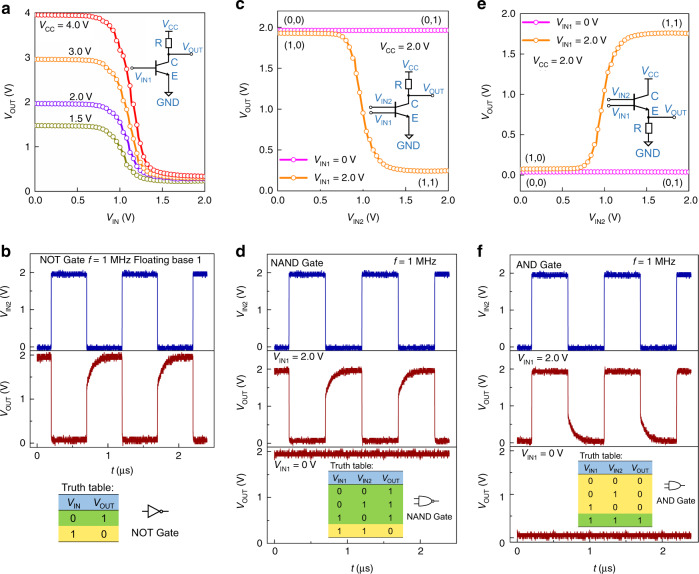
Static and dynamic characteristics of logic circuits realized by OPDBTs. **a**, **c**, **e** Circuit diagrams and static voltage transfer characteristics of an inverter (**a**), a NAND gate logic circuit (**c**), and an AND gate logic circuit (**e**). **b**, **d**, **f** Dynamic characteristics and truth tables of the above corresponding logic circuits. The resistive load inverter is realized by an OPDBT and a 400 kΩ external resistor. GND represents the ground.

By integrating a dual-base transistor with a resistive load, a logic inverter, NAND gate and AND gate circuits are realized experimentally, as illustrated in Fig. 5. The circuit diagram, static and dynamic voltage transfer characteristics (VTCs), and truth table of a resistive load inverter composed of a 400 kΩ external resistance and an OPDBT are shown in Fig. 5a, b for different supply voltages, respectively. In this configuration, only one of the base electrodes of OPDBTs is employed while the other one is kept floating. The VTCs are fine-tuned by changing *V*_CC_ and the highest voltage gain (d*V*_OUT_/d*V*_In_) is 14 as the input voltage varies from 0 to 2.0 V (see Supplementary Fig. 12). Besides the exceptionally small supply voltage, this gain is among the best values reported for unipolar inverters based on organic transistors^48,49^. The dynamic performance of the inverter is evaluated by applying a square-wave input with a frequency (*f*) of 1 MHz and an amplitude (*V*_IN_) of 2.0 V. It can be seen that the inverter operates well at 1 MHz. Presumably, the capacitance in OPDBTs may limit the dynamic response of the inverter^50^. Thus, OPDBTs can work well in the MHz region. A good performance and the highest voltage gain of 23 is also obtained for inverters with a depletion load as shown in Supplementary Fig. 13. In this case, the base of a second OPDBT is connected to the output of the inverter while the voltage of base1 is swept again between 0 and 2.0 V (*V*_CC_ = 2.0 V). Thus, the logic inverters can be simply implemented by using OPDBTs with one base input.

In Fig. 5c, d, the NAND gate operation is realized with an OPDBT as a function of base2 input voltage (*V*_IN2_) and fixed base1 voltage (*V*_IN1_ = 0, 2.0 V), under a supply voltage of 2.0 V. While, Fig. 5e, f show the static and dynamic VTCs of an AND gate when the base1 voltages (*V*_IN1_) are fixed at 0 and 2.0 V and the base2 voltage (*V*_IN2_) is swept from 0 to 2.0 V under a supply voltage of *V*_CC_ = 2.0 V. The VTCs in Fig. 5c, e exhibit well high and low states which are the result of the steep transfer curves of the OPDBTs in the logics. From the dynamic characteristics of Fig. 5d, f, NAND gate and AND gate logic circuits can operate well as expected at 1 MHz.

We conclude that logic circuits can be easily realized with our OPDBTs by using two base inputs simultaneously. Unlike conventional unipolar NAND/AND gates consisting of at least two transistors, we implement the NAND/AND gate by using only one vertically stacked dual-base transistors. The independent base control enables the transistor to work at different states by tuning the voltages of two bases (two inputs). OPDBTs with two inputs simplify the fabrication of logic circuits without compromising performance and the reduction of the total number of transistors offers great advantages for integrated circuits. Hence, since OPDBTs represent a compact and technologically simple hardware platform, they offer excellent application perspectives of vertical-channel organic transistors in complex logic circuits. Furthermore, since OPBTs are the organic transistors with the highest operation frequency reported today, further studies and device optimization might enable OPDBTs to be used in high-frequency logic circuits by further study.

## Discussion

In summary, a device concept of organic dual-base transistors has been demonstrated. By employing this vertical-channel dual-base structures in organic transistors, we can easily control the *V*_th_ which is a main requirement for designing efficient logic circuits. Excellent electrical performance is obtained for this transistor design, e.g., a high on-current density of 1.54 A cm^−2^, a large current gain of 9.2 × 10^5^, corresponding to a high transmission value of 99.998%, which is almost comparable to single-base OPBTs reported. The numerical TCAD simulation of the structure will help to study structure engineering towards optimizing the performance of OPDBTs. The good agreement between experimental data and numerical simulation confirms the viability of the proposed device design with a channel length in the range of only a few hundreds of nanometers. The functionality enhanced device structure enables to design logic circuits with just one single transistor. To prove the advantage and feasibility of the design, which are investigated as building blocks of the large-scale circuits, we also demonstrate logic gates including inverters, AND gates, and NAND gates which are operating at a voltage as low as 2.0 V.

## Methods

### Device fabrication

The OPDBTs presented are fabricated in a single chamber UHV-tool and one glass substrate previously cleaned with N-Methylpyrrolidone, distilled water, ethanol, and Ultra Violet Ozone Cleaning System. By using thermal vapor deposition at high vacuum (*p* < 10^−7^ mbar), the layer stack (Fig. 1a) is realized by subsequently depositing thin films through laser-cut, stainless steel shadow masks. The deposition system includes a wedge for realizing samples of different layer thicknesses in one run while other layers remain equal. The layer stack, evaporation rates and treatments of the OPDBTs are: Al 100 nm (2 Å s^−1^)/Cr 10 nm (0.1 Å s^−1^)/i-C_60_ 100 nm (1 Å s^−1^)/Al 15 nm (1 Å s^−1^)/15 min oxidation in ambient air/i-C_60_ 60 nm (1 Å s^−1^)/Al 15 nm (1 Å s^−1^)/15 min oxidation in ambient air/i-C_60_ 100 nm (1 Å s^−1^)/n-C_60_ 20 nm (0.4 Å s^−1^) co-evaporating C_60_ with W_2_ (hpp)_4_ (purchased from Novaled AG, Dresden) using 1 wt.%/1 x or 2 x (perpendicular to each other) SiO 100 nm with a free stripe of 0.2 mm (1 Å s^−1^)/Cr 10 nm (0.1 Å s^−1^)/Al 100 nm (2 Å s^−1^)/encapsulation under nitrogen atmosphere (<1 ppm O_2_ and H_2_O) using UV cured epoxy glue and cavity glasses without UV exposure of the active area/annealing for 2 h at 150 °C on a hotplate in a nitrogen glove-box.

### Device characterization

Transistor characteristics are measured by using two Keithley 236 together with a Keithley 2400 SMU in a glovebox. For the impedance spectroscopy, an HP 4284 A LCR-Meter is used. For all the electrical characterizations, the measurement software SweepMe! (sweep-me.net) is used. The optical image is taken by optical microscopy. The scanning electron microscope (SEM) images are captured using a Zeiss Gemini SEM 500. TEM measurements are carried out with a Libra200 (Carl Zeiss Microscopy GmbH, Germany) operated at an acceleration voltage of 200 kV. The lamella for TEM is prepared by lift-out focused ion beam (FIB) technique in NEON40 FIB/SEM (Carl Zeiss Microscopy GmbH, Germany).

### TCAD simulation

Synopsys’ Sentaurus TCAD simulator has been used to simulate 3D structures. The device structure is created by a 3D Sentaurus structure editor. It incorporates advanced physical models and robust numeric methods and simulates the electrical behavior of semiconductor devices. The non-local tunneling model is used to include the contributions of the total tunneling current. Gaussian Density-of-States has been used to better represent effective DOS in disordered organic semiconductors. A simple constant carrier generation model is used.

## Supplementary information

Supplementary Information

## Data Availability

The data that support the plots within this paper and other findings of this study are available from the corresponding author upon reasonable request.
