# Supplementary material for: Vertical organic permeable dual-base transistors for logic circuits
Source: Nat Commun. 2020 Sep 18;11:4725. doi: 10.1038/s41467-020-18576-5 (PMC7501854; doi:10.1038/s41467-020-18576-5)
Supplement: Supplementary file 1 — Supplementary Information [file 41467_2020_18576_MOESM1_ESM.pdf]

Supplementary Information

Vertical organic permeable dual-base transistors for logic circuits

Guo et al

Supplementary Figures

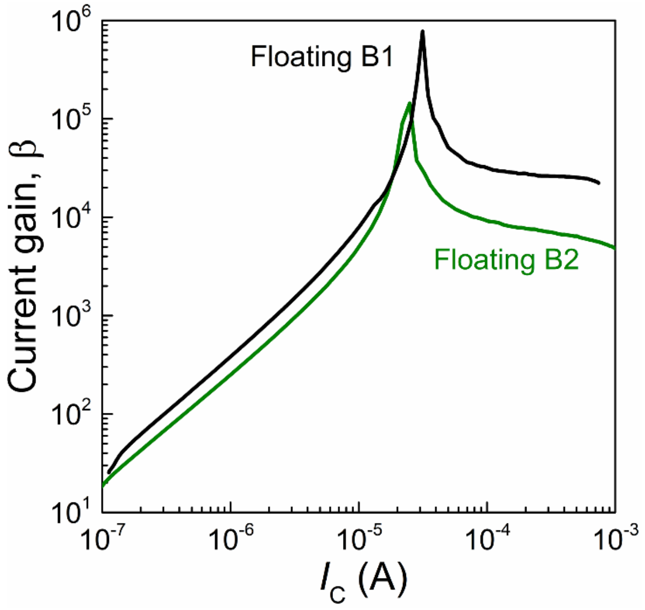

Supplementary Figure 1 | Current gain (β) of OPDBTs measured by floating one base. The maximum current gain of 7.7×10^5 and 1.4×10^5 when floating base1 and base2, respectively.

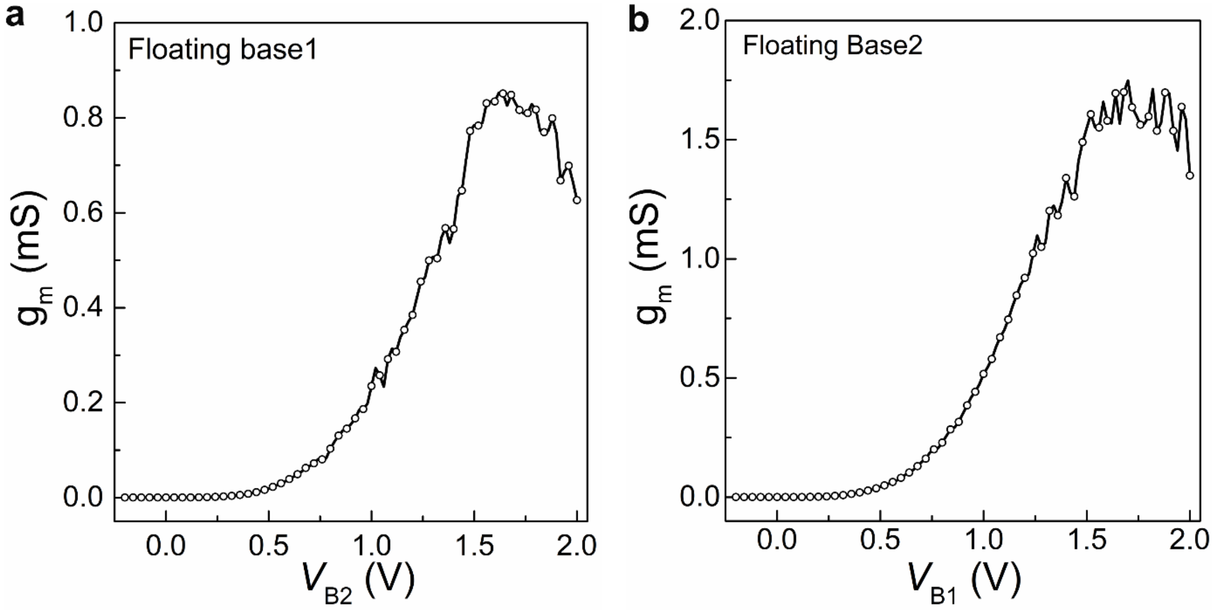

Supplementary Figure 2 | Transconductance (g_m) of OPDBTs measured by floating one base. **a-b**, Transconductance when floating base1 (**a**) and base2 (**b**) as a function of the base-emitter voltage. The maximum g_m value is 0.85 mS when floating base1 and 1.75 mS when floating base2.

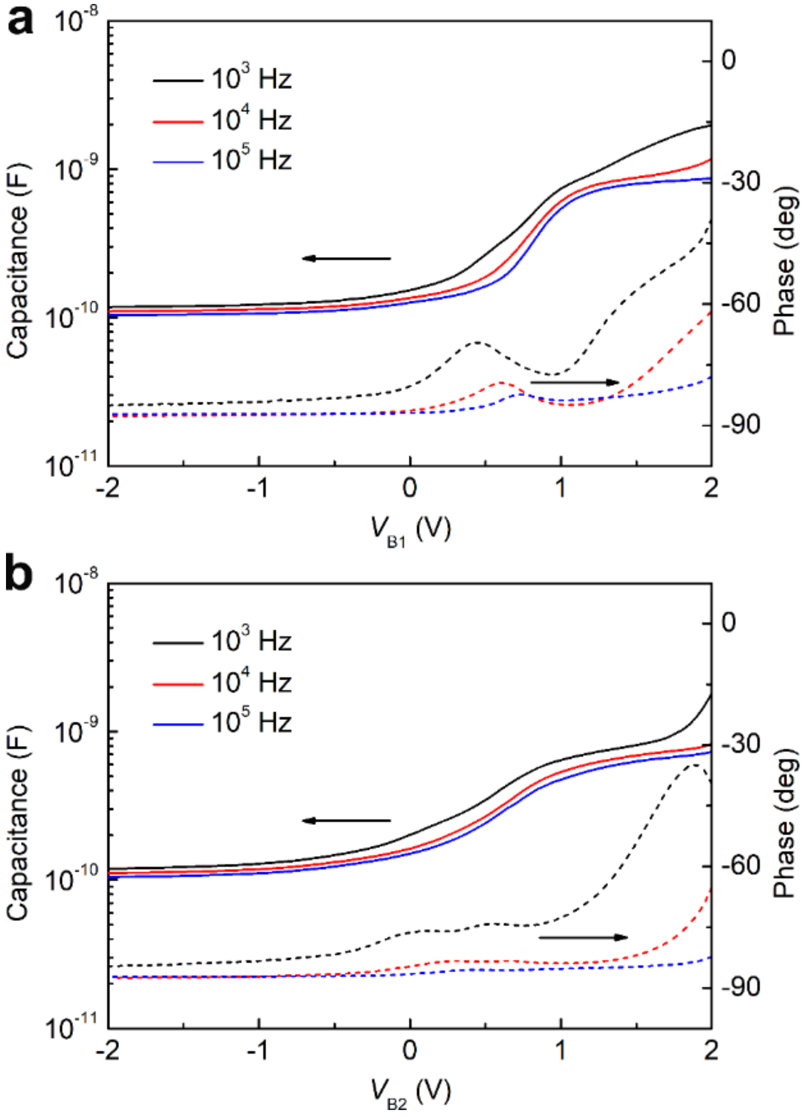

Supplementary Figure 3 | Capacitance and phase of OPBDTs. a, Capacitance and phase curves versus V_{B1} with different frequencies of 1 kHz, 10 kHz, and 100 kHz, respectively. **b**, Capacitance and phase curves versus V_{B1} with different frequencies of 1 kHz, 10 kHz, and 100 kHz, respectively. The phase stays close to -90°, indicating excellent insulating properties of the base oxide layer.

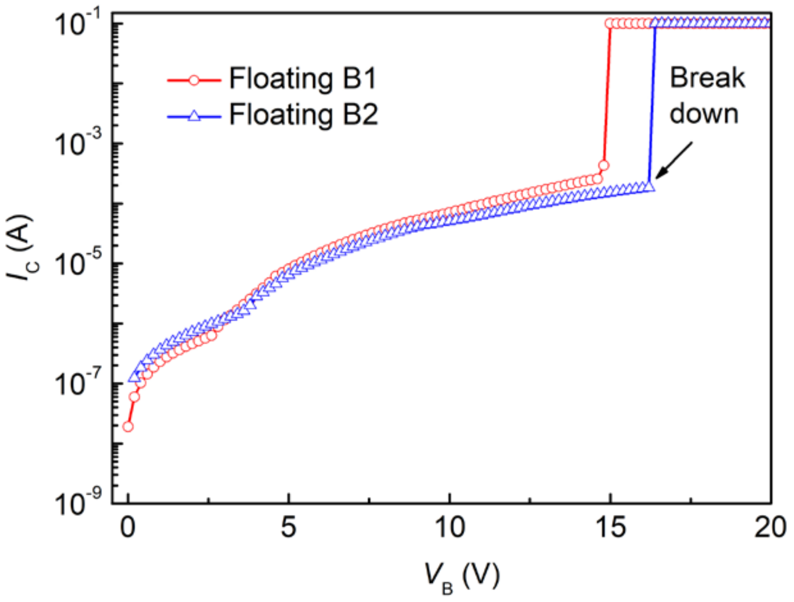

Supplementary Figure 4 | Breakdown voltages. OPDBTs withstand electric field between the base and collector electrodes.

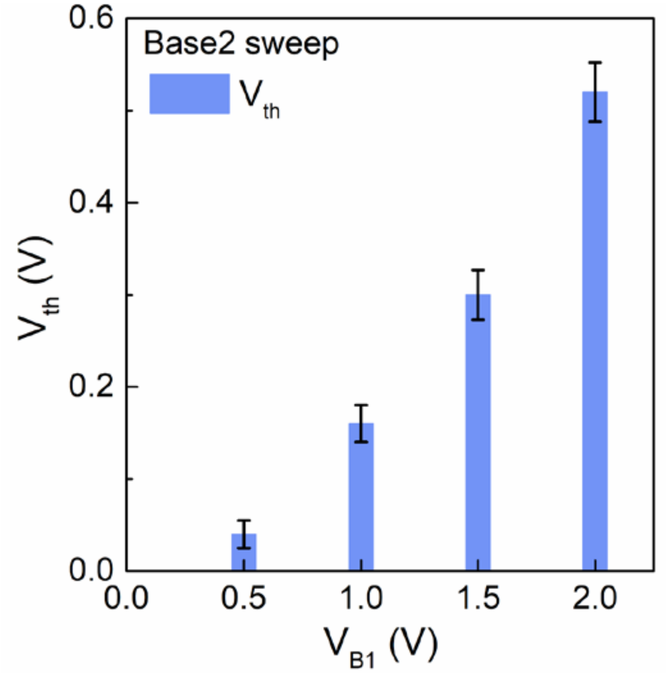

Supplementary Figure 5 | Threshold voltage shifts. The threshold voltages are dependent on the base1 bias during base2 sweep, error bars indicate the slight device-to-device variations measured over 61 devices.

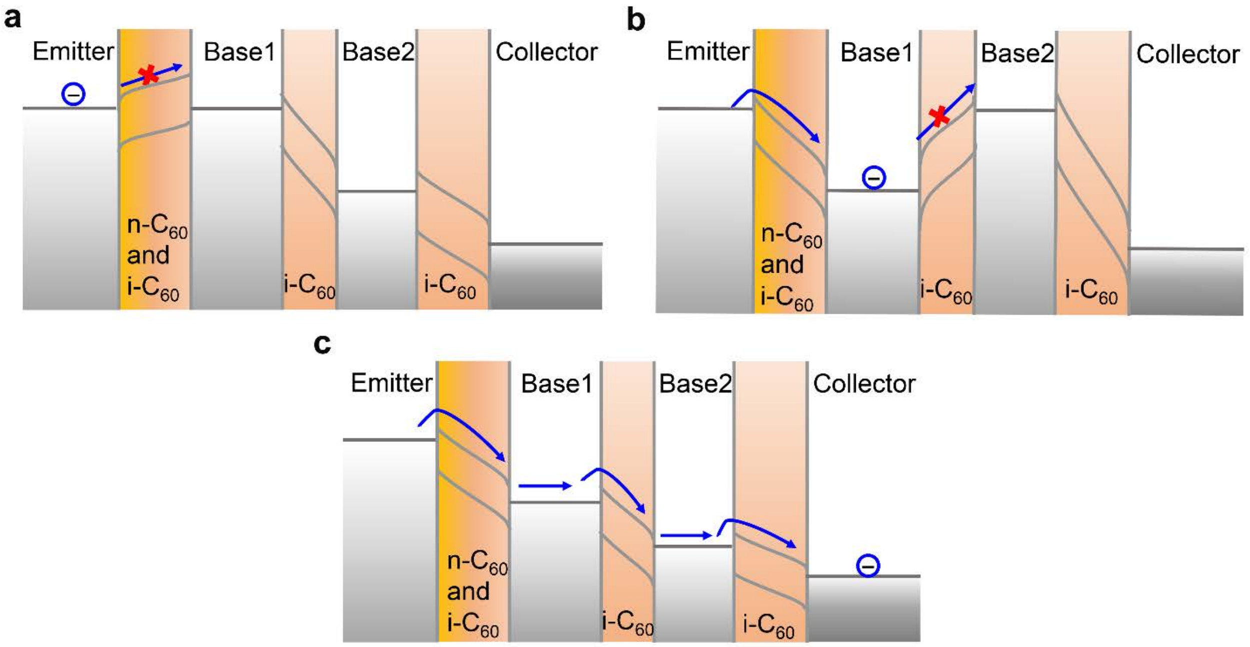

Supplementary Figure 6 | Energy band diagrams. a-**c**, Energy band diagrams of OPDBTs in off-state when base1 at low potential (**a**) and when base1 at high potential but base2 at low potential (**b**). **c,** Energy band diagram of OPDBTs in on-state (high base1 potential and higher base2 potential than base1), electrons can pass through the openings in both bases and reach the collector.

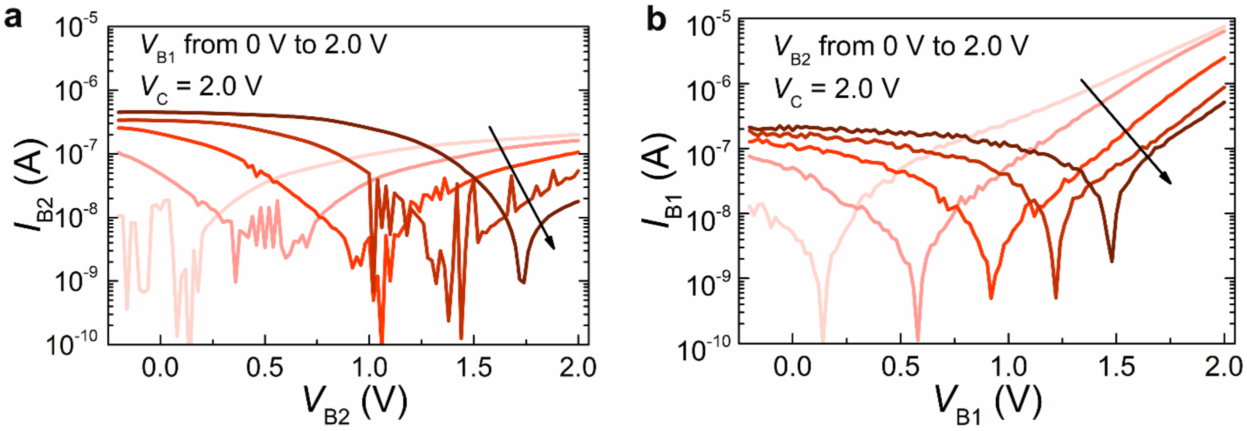

Supplementary Figure 7 | Base leakage currents. **a**, Base2 leakage current curves obtained during base2 sweep when V_C = 2.0 V and V_{B1} = 0, 0.5, 1.0 and 2.0 V, respectively. **b**, Base1 leakage current curves obtained during base1 sweep when V_C = 2.0 V and V_{B2} = 0, 0.5, 1.0 and 2.0 V, respectively.

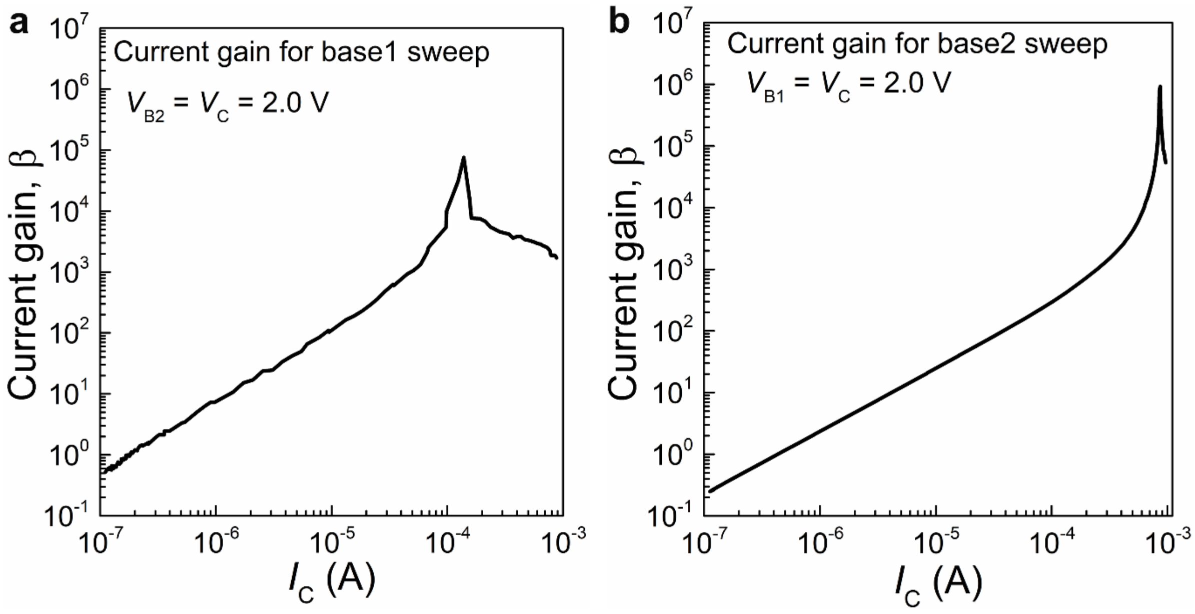

Supplementary Figure 8 | Current gain. **a**, Current gain of OPDBTs obtained during base1 sweep when V_{B2} = V_C = 2.0 V as a function of collector current. **b**, Current gain of OPDBTs obtained during base2 sweep when V_{B1} = V_C = 2.0 V as a function of collector current.

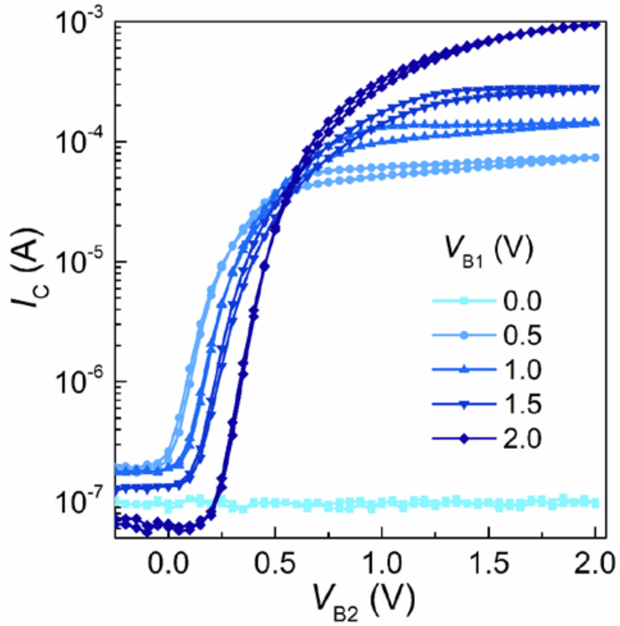

Supplementary Figure 9 | Hysteresis behavior. Transfer characteristics (forward and backward sweep) as a function of V_{B2} at different V_{B1} of 0, 0.5, 1.0, 1.5 and 2.0 V, respectively.

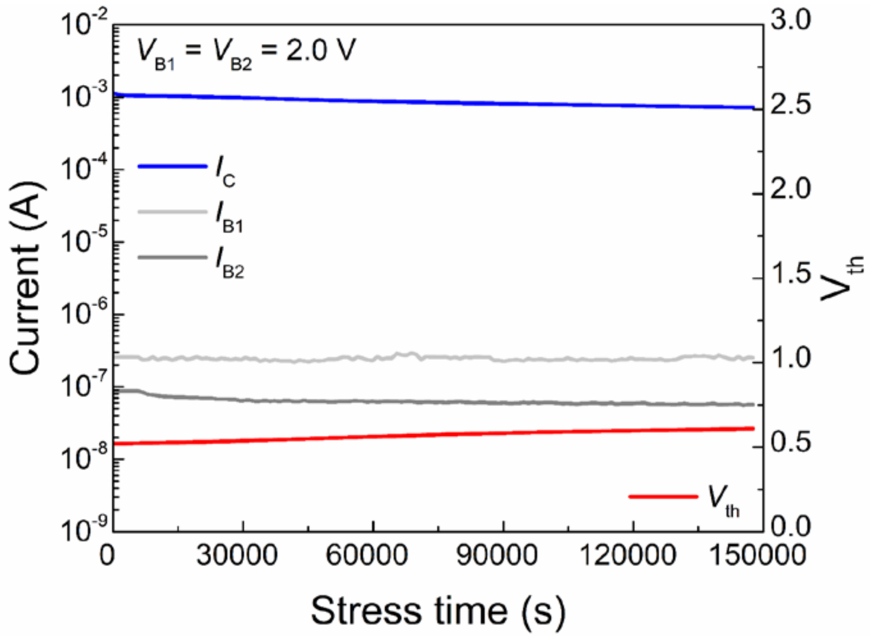

Supplementary Figure 10 | Bias stress stability. Bias stress measurement of OPDBTs when $V_C = V_{B2} = V_{B1} = 2.0$ V.

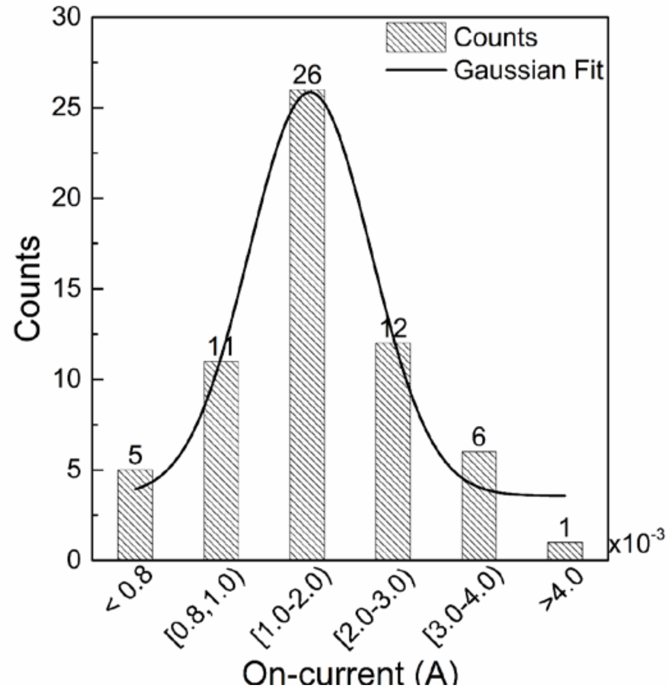

Supplementary Figure 11 | Device-to-device reproducibility. Maximum on-current distribution of 61 OPDBT devices from different batches.

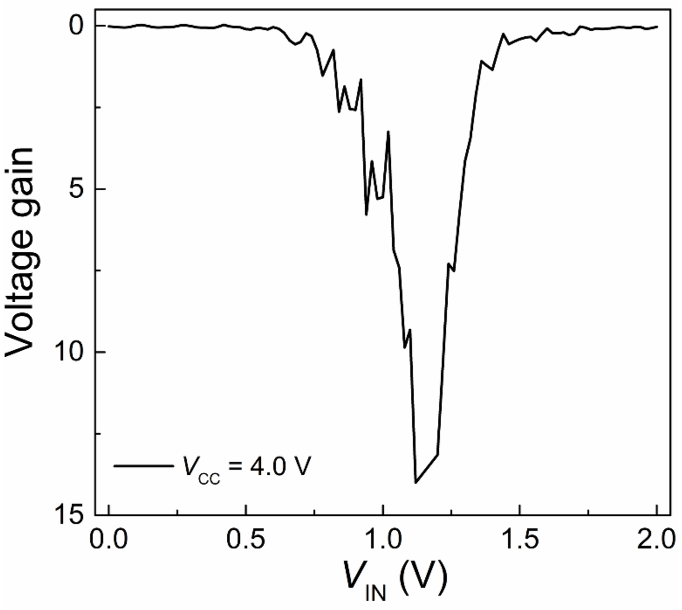

Supplementary Figure 12 | Voltage gain. Voltage gain of the resistive load inverter when supply voltage V_{CC} = 4.0 V.

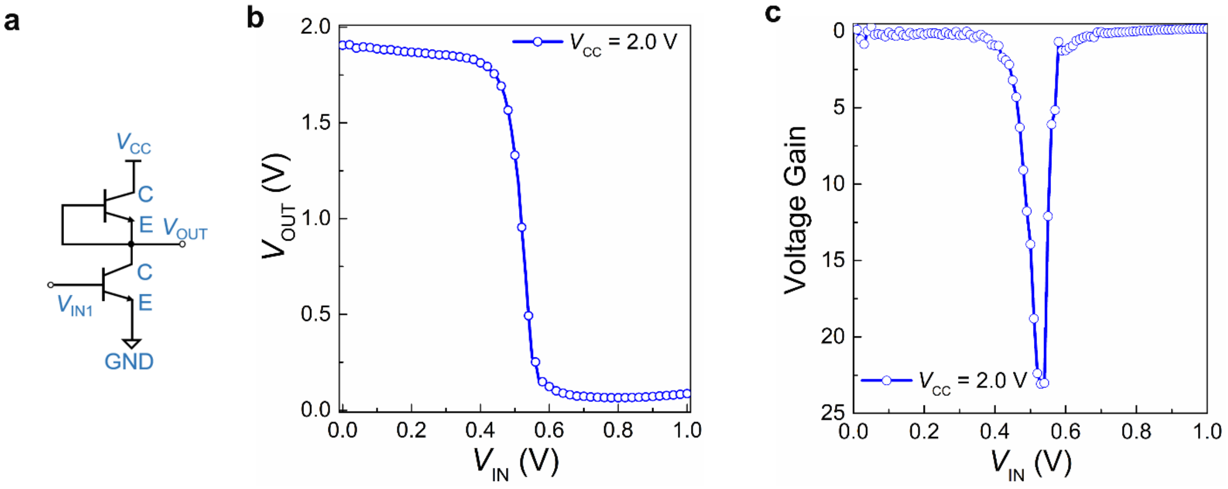

Supplementary Figure 13 | Inverter with depletion load. **a**-**c**, Circuit diagram (**a**), experimental voltage transfer characteristics (**b**), and voltage gain (**c**) of an inverter realized by an OPDBT and a depletion load as swept by base1 bias when V_{CC} = 2.0 V.

Supplementary Tables

Supplementary Table 1. **Summary of OPDBTs measured by floating one base**. Transmission value, on-current density, on-off ratio, threshold voltage (V_{th}), transconductance ($g_{m.max.}$), subthreshold swing (SS) and current gain ($\beta_{max.}$) of OPDBTs measured by floating one base.

	Transmission	On-current density (A cm^{-2})	On/off ratio	V_{th} (V)	$g_{m.max.}$ (mS)	SS (mV dec^{-1})	$\beta_{max.}$
**Floaing base2**	99.965% V_C=V_{B1} =2.0 V	2.25 V_C=V_{B1} =2.0 V	1.6 × 10^4	0.4	1.75	200	1.4 × 10^5
**Floating base1**	99.996% V_C=V_{B2} =2.0 V	1.19 V_C=V_{B2} =2.0 V	6.6 × 10^3	0.4	0.85	263	7.7 × 10^5

Supplementary Table 2. **Summary of OPDBTs**. Transmission value, on-current density, on-off ratio, threshold voltage (V_{th}), transconductance ($g_{m.max.}$), subthreshold swing (SS) and current gain ($\beta_{max.}$) of OPDBTs.

	Transmission	On-current density (A cm^{-2})	On/off ratio	V_{th} (V)	$g_{m.max.}$ (mS)	SS (mV dec^{-1})	$\beta_{max.}$
**Base2 sweep V_{B1} = 0.0 V**	52.649% V_C=V_{B2} =2.0 V	3.59×10^{-4} V_C=V_{B2} =2.0 V	1.5	-	-	-	-
**Base2 sweep V_{B1} = 0.5 V**	99.895% V_C=V_{B2} =2.0 V	0.248 V_C=V_{B2} =2.0 V	7.5 × 10^2	0.04	0.14	130	-
**Base2 sweep V_{B1} = 1.0 V**	99.977% V_C=V_{B2} =2.0 V	0.749 V_C=V_{B2} =2.0 V	2.2 × 10^3	0.16	0.44	136	-
**Base2 sweep V_{B1} = 1.5 V**	99.993% V_C=V_{B2} =2.0 V	1.2 V_C=V_{B2} =2.0 V	4.0 × 10^3	0.30	0.72	126	-
**Base2 sweep V_{B1} = 2.0V**	99.998% V_C=V_{B2} =2.0 V	1.544 V_C=V_{B2} =2.0 V	8.0 × 10^3	0.52	0.95	152	9.2 × 10^5
**Base1 sweep V_{B2} = 0.0 V**	3.979% V_C=V_{B1} =2.0 V	4.98×10^{-4} V_C=V_{B1} =2.0 V	1.9	-	-	-	-
**Base1 sweep V_{B2} = 0.5 V**	68.159% V_C=V_{B1} =2.0 V	0.022 V_C=V_{B1} =2.0 V	1.1 × 10^2	0.68	0.02	304	-
**Base1 sweep V_{B2} = 1.0 V**	98.505% V_C=V_{B1} =2.0 V	0.260 V_C=V_{B1} =2.0 V	1.3 × 10^3	0.82	0.25	251	-
**Base1 sweep V_{B2} = 1.5 V**	99.826% V_C=V_{B1} =2.0 V	0.809 V_C=V_{B1} =2.0 V	4.2 × 10^3	0.90	0.95	238	-
**Base1 sweep V_{B2} = 2.0 V**	99.942% V_C=V_{B1} =2.0 V	1.411 V_C=V_{B1} =2.0 V	7.9 × 10^3	0.92	1.76	235	7.6 × 10^4

Supplementary Table 3. TCAD Simulation Quantities. Parameters and units used for TCAD simulations.

**Quantity**	**Value/Unit**
Device Width W	1 μm
Device Length $L_E = L_C$	1 μm
OSC_{EB1} Thickness $T_{OSC(EB1)}$	100 nm
OSC_{B1B2} Thickness $T_{OSC(B1B2)}$	60 nm
OSC_{CB2} Thickness $T_{OSC(CB2)}$	100 nm
Oxide Thickness $T_{ox1} = L_{ox1}$	5 nm
Oxide Thickness $T_{ox2} = L_{ox2}$	5 nm
The Relative Permittivity of Oxide	7.8
Pinhole Diameter L_{Pin1}	4 nm
Pinhole Diameter L_{Pin2}	4 nm
Number of Pinholes L_{Pin1}	50 μm^{-2}
Number of Pinholes L_{Pin2}	50 μm^{-2}
Base/Emitter/Collector Thickness $T_{B/E/C}$	10 nm
Al Work Function	4.1 eV
Zero-Field Mobility μ_0	3 $cm^2V^{-1}s^{-1}$
Effective Density of States N_C	$1x10^{21}$ cm^{-3}
Width of the DOS Distribution σ_{DOS}	0.081eV
Energy Center E_c	0.1eV
Temperature T	300 K
LUMO (C_{60})	4 eV
Band Gap E_g (C_{60})	2.3 eV
HOMO (C_{60})	6.3 eV
Poole Frenkel Coefficient β	3
Poole Frenkel Coefficient γ	0
